# Glial Cells Missing 1 Regulates Equine Chorionic Gonadotrophin Beta Subunit *via* Binding to the Proximal Promoter

**DOI:** 10.3389/fendo.2018.00195

**Published:** 2018-04-26

**Authors:** Jordan E. Read, Victoria Cabrera-Sharp, Phoebe Kitscha, Judith E. Cartwright, Peter J. King, Robert C. Fowkes, Amanda M. de Mestre

**Affiliations:** ^1^Department Comparative Biomedical Sciences, Royal Veterinary College, London, United Kingdom; ^2^St. Georges Medical School, Molecular and Clinical Sciences Research Institute, University of London, London, United Kingdom; ^3^Centre for Endocrinology, William Harvey Research Institute, Queen Mary University of London, London, United Kingdom

**Keywords:** equine, trophoblast, placenta, equine chorionic gonadotrophin, glial cells missing 1, ETV1, ETV7, HOXA13

## Abstract

Equine chorionic gonadotrophin (eCG) is a placental glycoprotein critical for early equine pregnancy and used therapeutically in a number of species to support reproductive activity. The factors in trophoblast that transcriptionally regulate eCGβ-subunit (*LHB*), the gene which confers the hormones specificity for the receptor, are not known. The aim of this study was to determine if glial cells missing 1 regulates *LHB* promoter activity. Here, studies of the *LHB* proximal promoter identified four binding sites for glial cells missing 1 (GCM1) and western blot analysis confirmed GCM1 was expressed in equine chorionic girdle (ChG) and surrounding tissues. Luciferase assays demonstrated endogenous activity of the *LHB* promoter in BeWo choriocarcinoma cells with greatest activity by a proximal 335 bp promoter fragment. Transactivation studies in COS7 cells using an equine GCM1 expression vector showed GCM1 could transactivate the proximal 335 bp *LHB* promoter. Chromatin immunoprecipitation using primary ChG trophoblast cells showed GCM1 to preferentially bind to the most proximal GCM1-binding site over site 2. Mutation of site 1 but not site 2 resulted in a loss of endogenous promoter activity in BeWo cells and failure of GCM1 to transactivate the promoter in COS-7 cells. Together, these data show that GCM1 binds to site 1 in the *LHB* promoter but also requires the upstream segment of the *LHB* promoter between −119 bp and −335 bp of the translation start codon for activity. GCM1 binding partners, ETV1, ETV7, HOXA13, and PITX1, were found to be differentially expressed in the ChG between days 27 and 34 and are excellent candidates for this role. In conclusion, GCM1 was demonstrated to drive the *LHB* promoter, through direct binding to a predicted GCM1-binding site, with requirement for another factor(s) to bind the proximal promoter to exert this function. Based on these findings, we hypothesize that ETV7 and HOXA13 act in concert with GCM1 to initiate *LHB* transcription between days 30 and 31, with ETV1 partnering with GCM1 to maintain transcription.

## Introduction

Equine chorionic gonadotrophin (eCG) is a heterodimeric glycoprotein critical for early equine pregnancy. The primary function of eCG is the rescue of the corpus luteum and luteinization of secondary corpora lutea through binding to eLH/CG receptors (1, 2). As a direct consequence of this binding, a progesterone-rich environment necessary for early conceptus development is maintained (3). In other species, eCG also has a high affinity for FSH receptors (4, 5). As such, eCG extracted from pregnant mare sera is utilized therapeutically in cows and laboratory species to support follicle development and reproductive activity (6).

The source of eCG is the specialized terminally differentiated binucleate trophoblast cells of the chorionic girdle (ChG), and later the unique structures they form in the endometrium, termed the endometrial cups (7). Corresponding to the development of the ChG between days 30 and 36 of pregnancy, eCG can be detected in the sera of pregnant mares from around day 40. Beyond day 120 of pregnancy, eCG secretion diminishes corresponding to the destruction of the endometrial cups (8). While structural modifications that regulate eCG protein expression and activity are well defined (9), surprisingly little is known about the factors that initially induce and maintain the expression of eCG transcripts in equine trophoblast. In general, glycoprotein hormones are composed of an α-subunit; common to all glycoproteins in a species, and a β-subunit; which confers the hormones specificity for its receptor. In the equid, however, the β-subunit of equine luteinizing hormone (eLH) and eCG are encoded by a single gene (10, 11), yet demonstrate distinct tissue-specific expression profiles. Whereas pituitary specific direct transcriptional regulators of eLH, such as steroidogenic factor-1 (SF-1), have been investigated (12), to date, no placental specific regulator of LHB expression has been identified. In equine pregnancy, the β-subunit of eCG/LH is shown to demonstrate expression levels 100 times lower than that of the α-subunit (13), suggesting LHB is the limiting factor in the glycoproteins expression and subsequent activity.

Glial cells missing 1 (GCM1) is a transcription factor first identified in *Drosophila melanogaster* (14) and later defined as a member of the mammalian glial cells missing (GCM) family of proteins which possess a conserved DNA binding domain, a nuclear translocation sequence and two transactivation domains (15). In the human placenta, GCM1 is well known for its role in binding to the syncytin promoter (16), which in turn initiates fusion and formation of syncytiotrophoblast competent of secreting human CG (hCG) (17). Expression profiling of transcription factors in equine trophoblast showed *GCM1* mRNA expression is induced during ChG development (13). The functional role of GCM1 in equine trophoblast is not known. Based on the similar temporal expression patterns of *GCM1* and *LHB* (13), we hypothesized that GCM1 directly regulates *LHB via* binding to the *LHB* promoter.

The aim of this study was to determine (i) GCM1 protein expression in the ChG and (ii) whether GCM1 transcriptionally regulates *LHB* expression through direct binding to the *LHB* promoter.

## Materials and Methods

### Care of Animals

This study was carried out in accordance with the Animals (Scientific Procedures) Act 1986 guidelines set by the Home Office, United Kingdom. All protocols were approved by the Home Office and Ethics Committee of the Royal Veterinary College (PL70/6944). Eight mares (*Equus callabus*) aged between 3 and 7 years of Dartmoor or Welsh breed were maintained at the Royal Veterinary College in a paddock on grass and supplemented with hay over the winter. The reproductive cycle was manipulated, and pregnancies were established using standard artificial insemination and with ovulation induced using either 1,500 IU hCG (Chorulon, MSD Animal Health, Milton Keynes, UK) intravenously or 2.1 mg Ovuplant^®^ (Dechra Veterinary Products, Shrewsbury, UK) subcutaneously. Ovulation was confirmed (day 0) and then pregnancies were monitored biweekly and on the day of isolation using transrectal ultrasonographic evaluation of the reproductive tract. Only those conceptuses confirmed to have a normal growth rate and normal anatomical development were included in the study.

### Tissue Collection

Conceptuses were recovered by nonsurgical uterine lavage between days 27 and 34 of pregnancy, using established methods (18). Conceptuses were microdissected into ChG, allantochorion (ALC), chorion, yolk sac (YS), bilaminar omphalopleure, and fetus, and tissues immediately snap-frozen in liquid nitrogen.

### Construction of *LHB* Promoter Constructs, GCM Expression Vectors, and Transient Transfection of Immortalized Cell Lines

The 2,500-bp *LHB* promoter sequence, obtained from ENSEMBL 76 genome browser, was annotated with predicted GCM1-binding sites using Match software (http://gene-regulation.com) based on TRANSFAC^®^ Public 6.0. Truncated promoter constructs of specific lengths were constructed *via* PCR from equine genomic DNA, using primers designed with 5′ Mlu1 and 3′ Xba1 restriction sites. Primer sequences are available in Table S1 in Supplementary Material. PCR amplification was carried out using 15 ng of gDNA, in a 20-µL reaction, composed of: 10× PCR buffer, 0.2 mM each deoxynucleotide triphosphate, 1.5 mM MgCl (Invitrogen), 0.25 µM each primer, and 1.25 µL recombinant Taq DNA polymerase (Invitrogen), using Cycling parameters: denaturation for 2 min at 94°C, 35 cycles of 30 s at 94°C, 30 s at 59°C, and 1 min at 72°C, and a final extension step of 10 min at 72°C. PCR products were visualized by electrophoresis on a 1% (wt/vol) agarose gel. PCR products were purified, cloned, and sequenced to confirm specificity. Promoter products were inserted to the pGL3-basic expression vector, through digest of PCR product and vector with Mlu1 and Xba1 and subsequent ligation. Vectors were confirmed by Sanger sequencing. For mutation of the proximal two GCM1-binding sites, gBlocks^®^ gene fragments (Integrated DNA Technologies) were designed with a 2-bp mutation introduced to the site of interest and were cloned into expression vectors using 5′ Mlu1 and 3′ Xba1 restriction sites as described above.

A GCM1 expression vector was constructed *via* PCR amplification of the full length GCM1 gene from day 34 equine ChG cDNA and PCR conditions described above. GCM1 cDNA PCR products were cloned into the pCMV-myc expression vector (gift from Dr. Steve Allen, Royal Veterinary College) *via* subcloning into the Zero Blunt II^®^ TOPO^®^ vector (Invitrogen) *via* the manufacturers’ instructions. The pCMV-Myc vector and Zero Blunt II^®^ TOPO^®^-GCM1 were subjected to digest with XbaI and KpnI restriction enzymes and subsequent ligation with T4 DNA ligase.

Immortalized cell lines, BeWo Choriocarcinoma (BeWo) and COS7, were cultured in Dulbecco’s modified Eagle’s-medium (DMEM) supplemented with 10% fetal bovine serum, penicillin–streptomycin and l-glutamine, at 5% CO_2_ at 37°C. Cells were transiently transfected using Lipofectamine 2000 transfection reagent (Invitrogen) *via* the manufacturers’ instructions. Cells were transfected in 24-well plates, at a confluency of 80%, with each of four *LHB* promoter inserts or mutant promoter constructs, alone or in combination with concentrations of pCMV-myc-eqGCM1 (0–150 ng), with DNA input controlled with an empty control pCMV-myc vector. Renilla was co-transfected as an internal control at a concentration of 0.05 ng per well. Twenty-four hours post-transfection (selected following a preliminary time course experiment), cell lysates were harvested and promoter activity was measured using the Dual-luciferase^®^ Reporter Assay (Promega, USA), as per the manufacturer’s instructions.

### Culture Primary ChG Trophoblast

Chorionic girdle trophoblast cells were isolated and cultured from day 34 ChG tissue obtained from conceptuses as mentioned in Section “Tissue Collection.” In DMEM, ChG trophoblast cells were gently removed from basement membrane and underlying avascular mesodermal cell layer and cultured as previously described (18).

### RNA Isolation and cDNA Synthesis

Total RNA was isolated from snap-frozen equine ChG and chorion tissues, using an RNeasy kit (Qiagen) as per the manufacturer’s instructions. RNA (500 ng) was DNase I-treated (Invitrogen), and first-strand cDNA synthesis was performed using Moloney murine leukemia virus reverse transcriptase (US Biochemical Corp) as per the manufacturer’s guidelines.

### Quantitative RT-PCR

Quantitative RT-PCR of equine *LHB, GCM1*, or the housekeeper gene Succinate Dehydrogenase Complex, Subunit A, Flavoprotein (*SDHA*) (13) was carried out using SYBR Green chemistry (KAPA SYBR FAST Universal qPCR kit; KAPA Biosystems) with a C-1000 thermal cycler and CFX-96 Real time system (Bio-Rad Laboratories), in a total volume of 20 µL. Cycling conditions were: 38 cycles of 30 s at 95°C, 30 s at 60°C, and 20 s at 72°C. Post amplification, a melting curve was run from 60 to 95°C. Expression of *LHB* and *GCM1* mRNA in days 27–34 ChG and chorion tissues was calculated relative to day 27 ChG expression, using the Pfaffl method (19), taking into account the efficiency of the reaction for each gene and normalizing to *SDHA* expression. Primer sequences and reaction efficiencies can be found in Table S2 in Supplementary Material.

### Western Blot Analysis of Protein Expression

Protein was extracted from day 34 equine conceptus tissues by grinding and lysing on ice in lysis buffer, composition: 150 mM sodium chloride (Sigma), 1.0% Nonidet P-40 (Sigma), 0.5% sodium deoxycholate (Sigma), 0.1% sodium dodecyl sulfate (SDS, Sigma), 50 mM Tris (Sigma), pH 8.0, 1 mM phenylmethylsulfonyl fluoride (PMSF, Sigma). Protein was extracted from BeWo cells and COS7 cells, untransfected or transfected with pCMV-myc-GCM1 expression vector, using 50 µl lysis buffer, composition: 2% SDS, 2 M Urea (Sigma), 8% sucrose (Sigma), 20 mM sodium β-glycerophosphate (Sigma), 1 mM sodium fluoride (NaF, Sigma), and 5 mM sodium orthovanadate (Na_2_VO_4_, Sigma). Cells were scraped from wells and protein extracted using a Qiashredder column (Qiagen). Protein concentrations were determined using Bradford assay (Bio-Rad Laboratories). A total of 50 µg of protein per well was loaded and separated by SDS-PAGE on a 10% (wt/vol) polyacrylamide gel before being transferred to a Polyvinylidene fluoride membrane *via* wet transfer. Following activation in methanol and blocking for 1 h in Tris-buffered saline-Tween 20 (TBS-T) containing 5% (wt/vol) nonfat milk, membranes were incubated overnight at 4°C in a 1:1,000 dilution of rabbit anti-human GCM1 polyclonal antibody (Aviva Systems Biology), in TBS-T containing 5% (wt/vol) nonfat milk. Antibodies were optimized for specificity to horse GCM1 protein using COS7 cells transfected with equine GCM1 and untransfected control cells (data shown in results). Membranes were incubated for 2 h with a 1:10,000 dilution of goat antirabbit IgG secondary antibody conjugated to horseradish peroxidase (Sigma) in TBS-T containing 5% (wt/vol) nonfat milk. GCM1 protein was visualized by incubating membranes with ECL plus detection reagents (PerkinElmer) and exposure onto Hyperfilm ECL. As a loading control, membranes were stripped and reprobed for β-actin using a monoclonal mouse β-actin antibody (Sigma) at a dilution of 1:5,000. Densitometry analysis of western blots was carried out using ImageJ 1.47b software.

### Chromatin Immunoprecipitation (ChIP)

Chromatin ImmunoPrecipitation was carried out using the Chip-IT^®^ high sensitivity kit (Active Motif) *via* the manufacturers’ instructions. In brief, primary passage 1 equine day 34 ChG trophoblast cells were treated with paraformaldehyde to cross link protein–DNA complexes. Cells were ruptured *via* dounce homogenization and chromatin sheared using Micrococcal Nuclease (NEB) at a concentration of 1,400 U/μg chromatin. Sheared chromatin was incubated overnight at 4°C with an antihuman GCM1 antibody (Aviva Systems Biology) conjugated to protein G agarose beads and was subjected to column precipitation. Following reversal of crosslinks and DNA purification, qRT-PCR was carried out using primer pairs specified in Table S3 in Supplementary Material to assess enrichment of binding of GCM1 to predicted GCM1 binding sites in the *LHB* promoter. Data were expressed as enrichment of binding compared to a control region of the genome within the coding region of the LHB gene with no predicted GCM1 binding sites within 500 bp.

### Statistical Analysis

Statistical analysis was carried out using GraphPad PRISM^®^ V6.02 with the exception of gene expression data for *GCM1* and *LHB*, which was analyzed using SPSS (IBM Analytics). Luciferase assay data for basal promoter activity in BeWo and COS7 cells were subject to ordinary one-way ANOVA, with Dunnett’s multiple comparisons *post hoc* test. Luciferase assay data for transactivation studies were subject to ordinary two-way ANOVA, with Tukey’s multiple comparisons *post hoc* test. ChIP data were subject to ordinary one-way ANOVA, with Dunnett’s multiple comparisons *post hoc* test. Gene expression data for *GCM1* and *LHB*, from qRT-PCR, was performed using Linear Mixed-Effects Modeling in SPSS. In all cases, *p* ≤ 0.05–0.0001 was accepted as statistically significant.

All storage of biological material and laboratory protocols conformed to biosecurity and safety measures as set out in the Royal Veterinary College code of Good Laboratory Practice.

## Results

### GCM1 Expression in the Early Equine Conceptus

**Figure 1:**
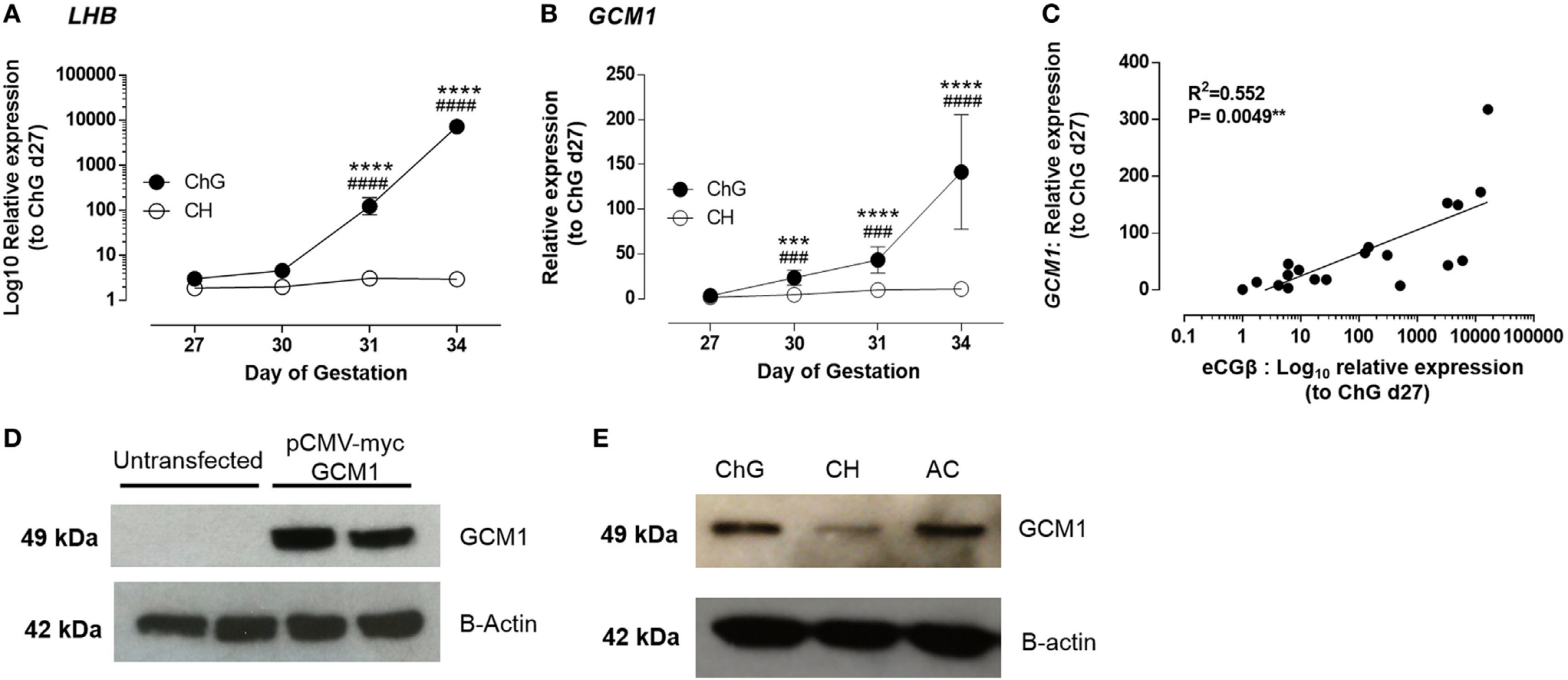
*GCM1* is expressed in equine trophoblast and expression correlates with *LHB*. **(A)** mRNA expression of *LHB* in ChG and chorion tissues at days 27, 30, 31, and 34 of pregnancy. **(B)** mRNA expression of *GCM1* in ChG and chorion tissues at days 27, 30, 31, and 34 of pregnancy. **(C)** Correlation of mRNA expression of *LHB* and *GCM1* in individual ChG tissues. ***p < 0.001, ****p < 0.0001 relative to day 27 ChG, ^###^*p* < 0.001, ^####^*p* < 0.0001 relative to CH at same time point (linear mixed-effects modeling, SPSS). **(D)** Glial cells missing 1 (GCM1) protein expression in untransfected COS7 cells and COS7 cells transfected with 250 ng of equine pCMV-myc-GCM1 expression vector. B-Actin expression was used as a loading control. **(E)** GCM1 and B-Actin protein expression in primary equine day 34 conceptus tissues: chorionic girdle (ChG), chorion (CH), allantochorion (ALC), and yolk sac (YS).

To confirm GCM1 as a candidate for *eCGB* gene regulation, the relative expression profiles of *LHB* and *GCM1* mRNA in the ChG and chorion (control) tissues from conceptuses at days 27, 30, 31, and 34 were quantified using qRT-PCR (*n* = 4 each time point and each tissue). This 7-day period in early equine pregnancy (of 340-day gestation period) represents the window of initiation of chorionic gonadotrophin expression in the mare and is analogous to early chorionic development in mouse and human placental development. No change in *LHB* and *GCM1* mRNA expression was observed in the chorion (Figures 1A,B). When compared with day 27, *LHB* mRNA expression in the ChG increased significantly by day 31 of pregnancy (93-fold, *p* < 0.0001) with further increased expression observed at day 34 (5,947-fold, *p* < 0.0001). Expression of *LHB* was 49-fold higher (*p* < 0.0001) in day 31 ChG and 5,808-fold higher (*p* < 0.0001) in day 34 ChG both compared to time-matched chorion (Figure 1A).

*GCM1* mRNA expression was highest within the developmental time-frame studied in day 34 ChG (100-fold, *p* ≤ 0.0001) when compared to day 27 ChG expression. A significant increase in *GCM1* expression was also observed at day 30 (11-fold, *p* = 0.002) and day 31 (24-fold, *p* < 0.0001) compared to day 27 ChG, thus preceding the increase in *eCGB* expression by 24 h. *GCM1* expression was also significantly increased compared to time matched chorion at all time points (day 30: 7.2-fold, *p* = 0.0013; day 31: 10.1-fold, *p* = 0.0009; day 34: 14.7-fold, *p* < 0.0001) (Figure 1B). In individual ChG tissues, *LHB* and *GCM1* mRNA expression was correlated with a correlation value *R*^2^ = 0.552 (*p* = 0.0049) (Figure 1C).

Western blot analysis was used to confirm GCM1 protein expression in equine ChG. First, to validate that the antihuman GCM1 monoclonal antibody was also able to detect the equine protein, COS-7 cells were transfected with an equine GCM1 expression vector (pCMV-myc eqGCM1). Following transfection, COS-7 cells expressed equine GCM1 protein as shown by a single band of expected size, 49 kDa (Figure 1D). This band was not observed in control untransfected COS-7 cells. GCM1 protein expression in day 34 ChG was subsequently confirmed (Figure 1E), with expression also detected in the surrounding fetal membranes, ALC and YS (*n* = 3).

### GCM1 Transactivates the *LHB* Promoter

Interrogation of the proximal 2,500 bp of the *LHB* promoter sequence identified four potential binding sites for GCM1 (Figure S1 in Supplementary Material) all with 100% match of the known core binding sequence GCGG and a matrix similarity greater than 80%. Sites were located at positions (1) −87 to −98 (Mat.Sim. = 0.903, binding sequence 5′-agccTGCGGgtat-3′), (2) −124 to −136 (Mat.Sim. = 0.865, binding sequence 5′-gccaTGCGGgcat-31), (3) −779 to −791 (Mat.Sim. = 0.869, binding sequence 5′-ccgcTGCGGggcc-3′), and (4) −2055 to −2067 (Mat.Sim. = 0.957, binding sequence 5′-attcTGCGGgggg-3′) relative to the translational start site and predicted to bind to the negative strand of DNA.

**Figure 2:**
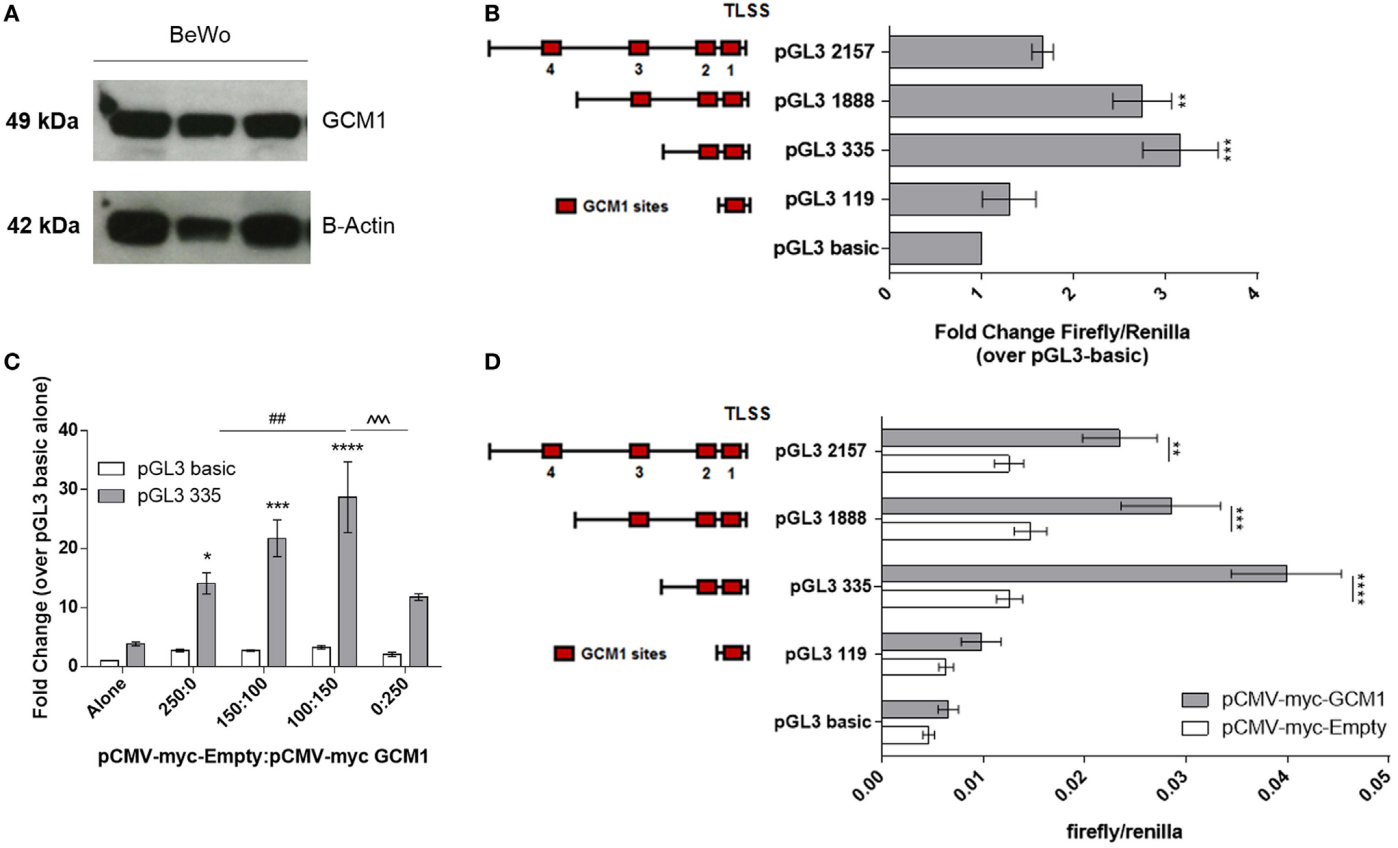
Glial cells missing 1 (GCM1) transactivates the *LHB* promoter. **(A)** Western blotting of protein extracted from human choriocarcinoma BeWo cells (*n* = 3) using an antihuman GCM1 monoclonal antibody (c-terminal). β-actin was used as a loading control. **(B)** BeWo cells were transfected with specific lengths of the *LHB* promoter in a pGL3-basic luciferase reporter vector (*n* = 3). Activity of promoter constructs was expressed as fold-change firefly/renilla compared to pGL3-basic. **p < 0.01, ***p < 0.001 (one-way ANOVA). **(C)** pGL3-basic and pGL3-335 were co-transfected with ratios of pCMV-myc-Empty: pCMV-myc-GCM1 to assess ability of GCM1 to drive promoter activity in COS7 cells (*n* = 3). *p < 0.05, ***p < 0.001, ****p < 0.0001 relative to pGL3-335 alone, ^##^*p* < 0.01 relative to pGL3-335 + 250 ng pCMV-myc-Empty, ^ˆˆˆ^*p* < 0.001 relative to pGGl-335 + 150 ng pCMV-myc-GCM1 (two-way ANOVA). **(D)** Specific lengths of the *LHB* promoter were transfected with 150 ng of empty pCMV-myc-Empty vector, or with 150 ng of pCMV-myc-GCM1 (*n* = 4). *p < 0.05, **p < 0.0.01, ***p < 0.001, ****p < 0.0001 (two-way ANOVA).

As equine trophoblast cell lines are not available, *in vitro* experiments to interrogate the activity of the *LHB* promoter were performed using human BeWo choriocarcinoma trophoblast cells. Western blotting confirmed the BeWo cells expressed GCM1 (*n* = 3) (Figure 2A). Bioinformatic analysis showed human and equine GCM1 protein sequences were 79% homologous and highly homologous in the DNA binding domain (94.5%) (Figure S2 in Supplementary Material). Truncated *LHB* promoter constructs as represented in Figure 2B were designed to encompass between 1 and 4 predicted GCM1-binding sites and transfected into BeWo cells. Luciferase assays assessing basal *LHB* promoter activity demonstrated that only the pGL3-335 and pGL3-1888 promoter constructs were active above background activity of pGL3-basic alone (*n* = 3) (Figure 2B).

In order to assess transactivation of the *LHB* promoter by GCM1, the COS7 cell line (which was shown in Figure 1D to not express endogenous GCM1) was co-transfected with *LHB* promoter constructs and our equine GCM1 expression vector (pCMV-myc-eqGCM1) or an empty vector (pCMV-myc). First, the optimal concentration of pCMV-myc-eqGCM1 was determined. Addition of a ratio of pCMV-myc-Empty 100:150 pCMV-myc-eqGCM1 was able to increase promoter activity of pGL3-335 26-fold (*p* = 0.016) over pGL3-335 alone (Figure 2C); however, addition of higher ratios of pCMV-myc-eqGCM1 was unable to further drive the promoter above the levels of 150 ng pCMV-myc-eqGCM1. Next, co-transfection of the four *LHB* promoter constructs with either the pCMV-myc-empty vector (150 ng) or pCMV-myc-eqGCM1 (150 ng) showed that GCM1 transactivated the pGL3-335 construct 3.1-fold (*p* < 0.0001) over pCMV-myc-Empty (Figure 2D). The pGL3-1888 construct (1.9-fold, *p* = 0.0002) and pGL3-2157 construct (1.8-fold, *p* = 0.004) were both significantly driven by pCMV-myc-eqGCM1 when compared with pCMV-myc-Empty. Addition of pCMV-myc-eqGCM1 did not drive activity of the 119-bp insert containing the most proximal site 1 GCM1-binding site.

### GCM1 Binds the *LHB* Promoter *via* Interaction With the Proximal GCM1 Binding Site

**Figure 3:**
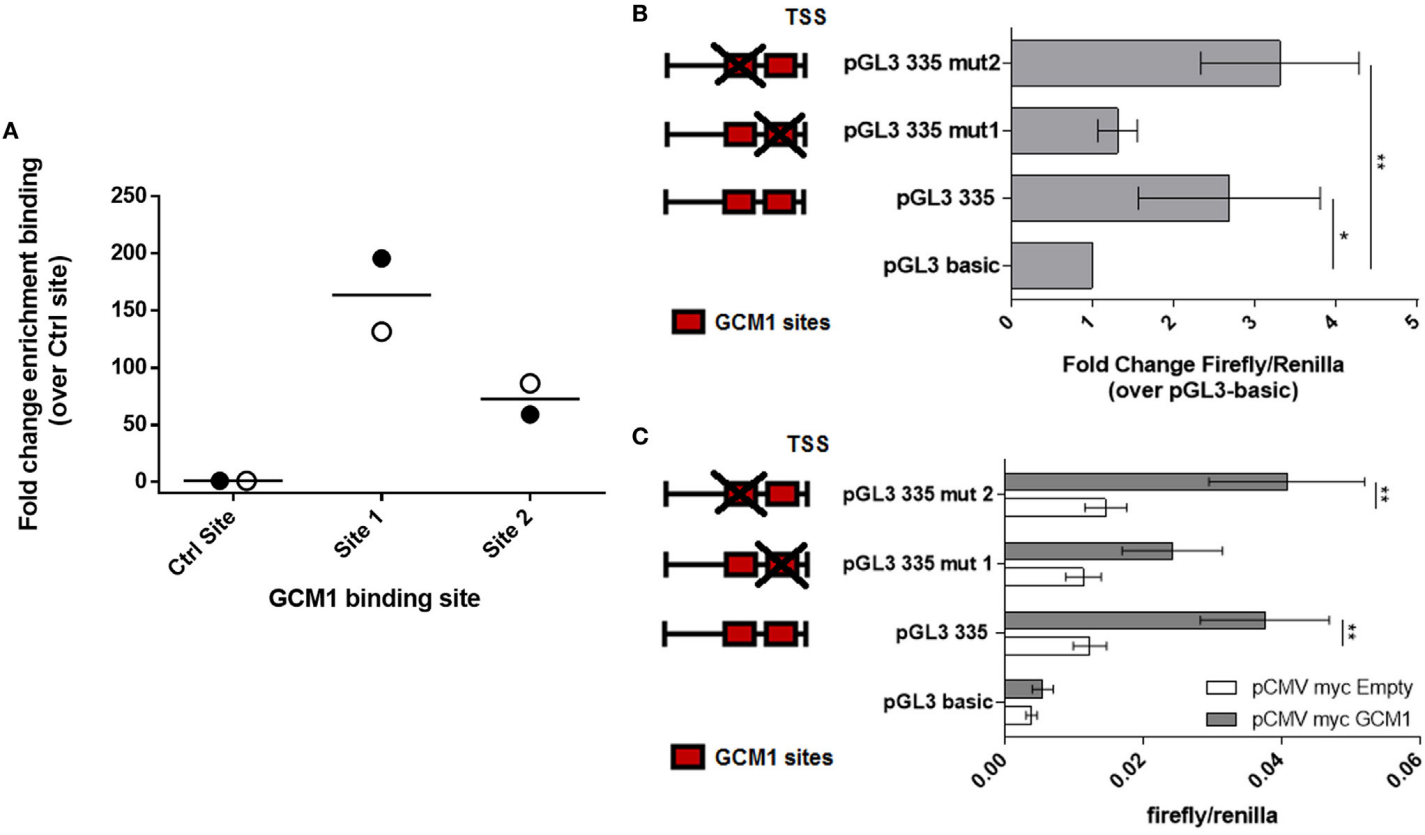
Glial cells missing 1 (GCM1) binds to the proximal *LHB* promoter in primary equine chorionic girdle (ChG) trophoblast cells and mutation of GCM1 site 1 results in loss of promoter activity. **(A)** Chromatin ImmunoPrecipitation was carried out on formaldehyde fixed chromatin complexes from passage 1 day 34 primary ChG trophoblasts, using an antihuman GCM1 antibody for immunoprecipitation. Binding of GCM1 to predicted GCM1-binding sites was expressed as fold-change enrichment over binding to a control region within the coding sequence of the *LHB* gene (*n* = 2 conceptuses). **(B,C)** The two GCM1-binding sites in the pGL3-335 promoter construct were mutated. Red boxes depict GCM1 binding sites. Red box with a cross depicts a mutated site. Wild-type pGL3-335 and its two mutant constructs, pGL3-335-mut1 and pGL3-335-mut2, were co-transfected **(B)** into BeWo cells (*n* = 3) **(C)** into COS7 cells, with 150 ng of pCMV-myc-GCM1 or pCMV-myc-Empty as a control (*n* = 4). Promoter activity was assessed by luciferase assay. *p < 0.05, **p < 0.01, ****p < 0.0001 [**(B)** one-way ANOVA, **(C)** two-way ANOVA].

In order to determine whether GCM1 drives *LHB* promoter activity through direct binding to one or more of the binding sites, ChIP was performed using formaldehyde fixed chromatin complexes from passage one day 34 primary equine ChG trophoblast cells (*n* = 2 conceptuses) and the previously validated cross reactive antihuman GCM1 antibody (Figure 1D) (Figure S3 in Supplementary Material). Quantitative RT-PCR was then carried out using primers designed against GCM predicted binding sites 1–3 and a control site within the coding region of the LHB gene with no predicted GCM1-binding sites within 500 bp. There was minimal binding in the control amplified region. Amplification of the promoter region flanking GCM1-binding site 1 identified enriched binding in this region when compared to the control region of the *LHB* gene (164-fold, *p* = 0.054, Figure 3A). There was modest enrichment of binding in GCM1 binding site 2 (Figure 3A). The PCR reaction for binding site 3 failed to amplify a product.

To confirm that GCM1 transactivates the LHB promoter *via* binding to site 1, a 2 base pair mutation was introduced into the core-binding site of GCM1 sites 1 and 2. Inserts were then transfected into BeWo cells to determine endogenous promoter activity (*n* = 3). Mutation of GCM1 binding site 1 rendered the pGL3-335 promoter inactive in BeWo cells, with no change in activity compared with pGL3 basic (Figure 3B). The pGL3-335-MUT2 construct, with disruption of binding site 2, remained significantly active above pGL3-basic with similar activity to the intact construct, pGL3-335 (Figure 3B). Inserts were then transfected into COS7 cells alone or with pCMV-myc-eqGCM1 to determine whether mutation of binding sites 1 and 2 disrupted the ability of GCM1 to transactivate the *LHB* promoter (*n* = 4). Consistent with the results in BeWo cells, pCMV-myc-eqGCM1 transactivated pGL3-335 and pGL3-335MUT2 but failed to significantly transactivate pGL3-335-MUT1 (Figure 3C).

### Other Transcription Factors Are Proposed to Regulate *LHB*

Results above indicate that GCM1 binds to GCM1 binding site 1 located within 119 bp of the translational start site but activity also required the upstream segment of the CGB promoter between −119 and −335. This suggested that another factor or factors may act in concert with GCM1. Recently, a number of new specific transcription factor-transcription factor interactions were identified including 14 proteins found to form DNA binding complexes with GCM1 (20). Next, we interrogated microarray data generated in the laboratory as part of another project (GEO: GSE113072) to determine whether these 14 GCM1 binding partners were differentially expressed in the ChG between days 27 and 34. Twelve of the genes were represented in the microarray with 7/12 genes differentially expressed (*n* = 4, fold-change > 2 relative to day 27 ChG, FDR *p* < 0.05) during ChG development: *ETV1, ETV7, HOXA13, PITX1, HOXB13, ONECUT2*, and *ELK3*.

**Figure 4:**
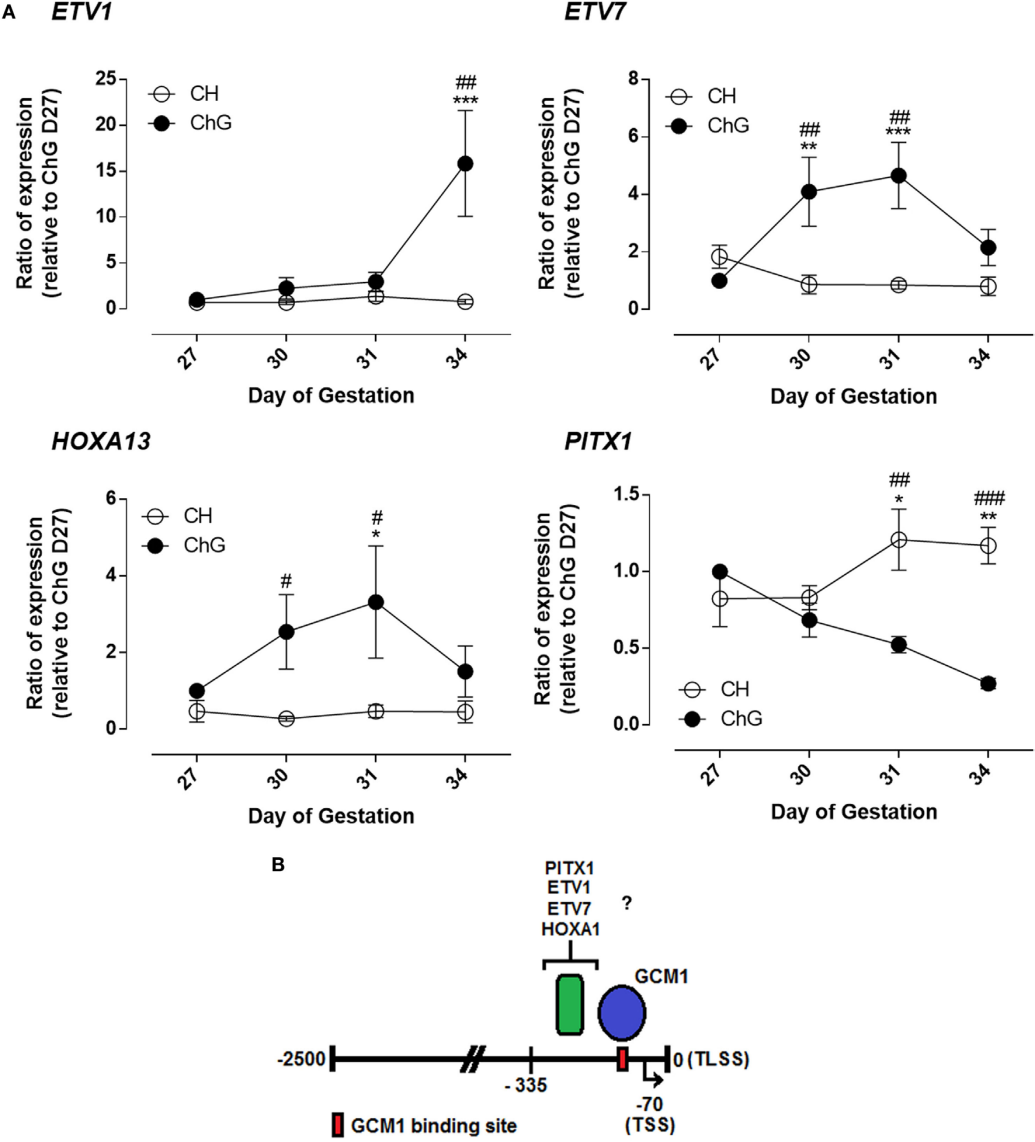
Glial cells missing 1 (GCM1) binding partners are differentially expression *in vivo* during differentiation of chorionic girdle (ChG) trophoblast cells. **(A)** mRNA expression of *ETV1, ETV7, HOXA13*, and *PITX1* in ChG and chorion tissues between days 27 and 34 of pregnancy, as determined by microarray analysis (*n* = 4). *p < 0.05, **p < 0.01, ***p < 0.001 relative to day 27 ChG, ^#^*p* < 0.05, ^##^*p* < 0.01, ^###^*p* < 0.001, relative to time matched chorion. **(B)** Schematic of the LHB promoter showing directly bound GCM1-binding site and region of potential binding by other transcription factors. TLSS is translational start site, TSS shown by black arrow is transcriptional start site. Numbers are relative to TLSS.

The four genes with the greatest magnitude of differential expression in the ChG between days 27 and 34 expression were *ETV1, ETV7, HOXA13*, and *PITX1* (Figure 4A). *ETV1* expression did not differ significantly over time or between tissues until day 34 of pregnancy. At day 34 of pregnancy *ETV1* expression was 9.4-fold greater in the ChG than day 27 ChG (*p* = 0.0008). *ETV7* expression rose significantly by 3.6-fold by day 30 of pregnancy in the ChG compared to day 27 ChG (*p* = 0.003). Expression remained transiently increased at day 31 of pregnancy (4.2-fold, *p* = 0.001) before returning to expression levels comparable to that at day 27 by day 34 (Figure 4A). *HOXA13* mRNA expression also increased significantly in the ChG at day 30, remaining transiently increased at day 31 and decreasing at day 34 to expression comparable to day 27 ChG. *PITX1* expression decreased in the ChG over the time course with expression 1.6-fold lower in the ChG at day 31 (*p* = 0.024) and 4.2-fold lower in the ChG at day 34 (*p* = 0.0019), compared to the day 27 ChG. Expression remained unchanged in the chorion for all four genes (Figure 4A).

## Discussion

Here, we show for the first time regulation of *LHB* appears to be under the control of direct binding of GCM1 to a GCM1-binding site immediately proximal to the transcription start site. Expression of GCM1 at the mRNA and protein level, combined with its confirmed correlation to *LHB* expression between days 27 and 34 of pregnancy in the ChG are all consistent with this role. Regulation of *LHB* by GCM1 is predicted, however, to require a co-operative binding of another transcription factor within the promoter region between 119 and 335 bp upstream of the TLSS. GCM1-binding partners, *ETV1, ETV7*, and *HOXA13* were found to be induced in the ChG around the time of initiation of *LHB* expression and as such are excellent candidates for this role. Figure 4B demonstrates schematically the understanding of *LHB* promoter regulation as described within this study. It is of note that developmental stages beyond day 34 may offer further insight into regulation of maintenance of eGC expression.

Glial cells missing 1 was first proposed as a candidate for regulation of *LHB* expression based on previous data obtained in the ChG (13), whereby GCM1 mRNA expression increased through pregnancy days 27–34, at the time of trophoblast differentiation and increase in eCG production. Expression data presented in this present study was fully supportive of these observations, confirming *GCM1* expression in the ChG increased significantly sevenfold by day 30 of pregnancy compared to day 27, thus preceding the increase in expression of *LHB* observed from day 31 of pregnancy. In addition, expression of *GCM1* and *LHB* in individual ChG tissues was found to be highly correlated. The early increase in *GCM1* expression, relative to the increase in *LHB* expression, and the correlation of expression profiles, is consistent with a role for GCM1 to transcriptionally regulate *CG/LHB*. This was strengthened further by bioinformatics analysis of the GCM1 protein, which confirmed equine GCM1 to be structurally similar to its human homolog, a known placental specific transcription factor (21, 22). Like human GCM1, the equine protein has a placental specific expression profile and contains the highly conserved N-terminal DNA binding domain, characteristic of all GCM family proteins (13, 23).

Prior to this study, translation of GCM1 into protein within the ChG had not been demonstrated. Data here showed that at day 34, GCM1 protein is expressed by the ChG, as well as in the surrounding conceptus tissues; the chorion and ALC. This is reminiscent of the protein expression profile observed in both human and mouse placenta. In the murine placenta, GCM1 expression is confined to clusters of trophoblast cells of the chorion and later to the labyrinth, formed upon fusion of the allantois to the chorion (24). The ChG of the equine placenta originates from cells of the chorion and sits in contact with the ALC (7, 25), therefore expression of GCM1 observed in these tissues is in agreement with the expression observed in the mouse and human placental counterparts.

Glial cells missing 1 exerts its regulatory effects upon the *LHB* promoter *via* direct binding to a GCM1-binding site located between 87 to 98 bp upstream of the TLSS. Through co-transfection studies, mutation of the most proximal GCM1-binding site to the TLSS in the *LHB* promoter was shown to ablate the ability of GCM1 to drive the proximal 335 bp *LHB* promoter; the promoter region shown to be most responsive to GCM1 *in vitro*. ChIP studies demonstrated direct binding of GCM1 to this proximal GCM1-binding site. The observation that GCM1 is a direct regulator of *LHB* is consistent with the known role for GCM1 as a regulator of *hCGB* (26). It is thought that production of hCG by the syncytiotrophoblast of the human placenta causes an increase in intracellular cAMP, thus activating the protein kinase A (PKA) signaling pathway, which leads to further trophoblast fusion and hCG production. Activation of the PKA signaling pathway causes phosphorylation and subsequent stabilization of GCM1, which has been shown to directly bind to the proximal promoter of the *hCGB* gene in a feedback loop (26). Therefore, it is possible, taking into account the findings of this study and recent identification of a possible syncytin homolog in equine placenta (27), that a similar feedback regulation may occur in the equine trophoblasts, potentially explaining the correlation between *LHB* and *GCM1* mRNA expression and the steep increase in expression of both at the mRNA level between days 31 and 34 of pregnancy.

Glial cells missing 1 is the first transcription factor identified to regulate the *LHB* subunit in the equine placenta. Although some placental specific regulators of the equine glycoprotein α-subunit have been identified, including ATF and TSEB (12), the only studies surrounding the equine β-subunit focus on pituitary specific expression of eLHB in human pituitary cell lines (12). SF-1 was found to regulate activity of the eLHβ promoter in gonadotrope cell lines, but to be non-essential to basal promoter activity. The site of initiation of transcription of hCGB is 366 bp upstream of that for hLHB (12), resulting in distinct promoters for pituitary LHβ and placental CGβ and, therefore, different regulatory elements. In the equid the site of initiation, are identical for both genes (12). With SF-1-binding sites predicted by Wolfe (12) to be within the 335 bp proximal promoter, it is plausible SF-1 could also be a co-regulator of LHB in the placenta but expression profiles for SF-1 and promoter activity need to be ascertained. Interestingly, these studies also found the β-subunit promoter to be inactive in the BeWo cell line (12), a finding contradictory to the data presented here. Within this study, the BeWo cell line was used as a model for equine trophoblast cells, based on the demonstration that the BeWo cells expressed endogenous GCM1, can be driven to differentiate to a multinucleate population and produce hCG in culture.

It is possible that GCM1 is not only responsible for regulation of *LHB* expression, but may act upon other gene promoters to promote ChG development between days 27 and 34 of pregnancy. In the human placenta GCM1 is proven to act as a powerful transcriptional regulator of syncytin, responsible for syncytialisation of trophoblast cells and subsequent hCG production (16, 28). Human GCM1, therefore, also acts as an indirect regulator of hCG production. Its role in syncytium formation is confirmed in mouse studies, whereby GCM1-null mice fail to form sufficient placental villi (29). It must not be overlooked that, although GCM1 is here proven to regulate *LHB* directly, a conserved role for GCM1 in regulation of trophoblast differentiation may also exist. Until recently, no equine homolog of syncytin had been identified in horse placenta, but recent studies have identified an endogenous retrovirus (ERV), with similar properties to syncytin in other trophoblast of the equine placenta (27). It is possible that GCM1, having been identified here as a direct transcriptional regulator of *CG/LHB*, may play an additional role in ChG development, through transcriptional regulation of ERV expression. Studies to assess the aforementioned ERV expression in the ChG and possible interaction of GCM1 would be required to address this hypothesis.

Data obtained to date are strongly indicative of a role for one or more co-factors interacting with GCM1 to regulate *LHB* expression. Although binding of GCM1 to the most proximal binding site to the TLSS was confirmed in immunoprecipitation studies, presence of only this binding site, within the 119 bp proximal promoter, was not sufficient for GCM1 transactivation of promoter activity. The enhanced promoter activity of the 335 bp promoter construct over the 119 bp promoter construct is suggestive of a key transcriptional activator within the promoter region between −119 and −335 bp relative to the TLSS. However, disruption of GCM1 binding to the predicted binding site within this region (Site 2) had little effect upon GCM1’s ability to drive *LHB* promoter activity. Driving of the *LHB* promoter by GCM1, both endogenously in BeWo cells and in transactivation studies in COS7 cells, was only significantly reduced by deletion of the most proximal GCM1-binding site, within the 119 bp promoter. GCM1 is, therefore, almost certain to require interaction with another transcription factor, or factors, which bind somewhere between 119 and 335 bp of the TLSS in order to exert its regulation upon the *LHB* promoter.

It is known that transcription factors often interact with other factors, in complexes, to alter DNA binding specificity or enhance transcription (30). Studies by Jolma et al. (20) identified a large number of transcription factors to interact with GCM1 in this way to bind and regulate gene promoters. The theory for existence of a co-regulator of *LHB* may be further supported by the basal activity of the 335 bp and 2,157 bp *LHB* promoter constructs in the COS7 cell line. COS7 cells were shown to express no GCM1 at the mRNA or protein level; therefore, another factor must be acting upon the promoter inserts to drive basal promoter activity in this cell line. Identification of other transcription factors which directly bind to and regulate activity of the *LHB* promoter is a major area of future study to increase our comprehension of eCG production by ChG trophoblast cells.

In summary, here we have demonstrated that GCM1 is a direct transcriptional regulator of *LHB* in ChG cells of the equine placenta. A number of key questions have arisen from the data, investigation of which is integral to a fuller understanding of the transcriptome of ChG trophoblast cells, ChG development, and eCG production. Further studies are required to determine the possible role of GCM1 binding partners play in *LHB* regulation.

## Ethics Statement

This study was carried out in accordance with the Animals (Scientific Procedures) Act 1986 guidelines set by the Home Office, United Kingdom. All protocols were approved by the Home Office and Ethics Committee of the Royal Veterinary College (PL70/6944).

## Author Contributions

AdM, JC, RF, and JR designed the study. JR, VC-S, PK, and PJK performed the experiments. JR and AdM analyzed the data and wrote the paper.

## Conflict of Interest Statement

The authors declare that the research was conducted in the absence of any commercial or financial relationships that could be construed as a potential conflict of interest.

## Supplementary Material

Figure S1 The CGB promoter contains four possible glial cells missing 1 (GCM1)-binding sites. The 2,500-bp *LHB* promoter sequence was obtained from ENSEMBL genome browser and Match software was used to identify GCM1 consensus binding sites with a core sequence match of 1 and matrix similarity >0.8. The four consensus-binding sites (underlined in red) for GCM1 are located at positions (1) −87 to −98 (2) −124 to −136 (3) −779 to −791 and (4) −2055 to −2067, relative to the translational start site. Arrow depicts transcriptional start site at −70 bp, and ATG depicts translational start site. Numbers on diagram are relative to translational start site.

Figure S2 Structure of the glial cells missing 1 (GCM1) family. Shown is human GCM1, located on chromosome 6. The coding region of glial cells missing spans exons 2 to 6 in the genomic sequence. Nucleotide position numbers show start and finish of coding region. The protein consists of a conserved N-terminal DNA binding domain, underlined in red on the cross-species alignments, a nuclear localization sequence and two C-terminal transactivation domains. The C-terminal region shows poor species conservation. Seven conserved cystein residues are shown with black arrows. * denotes conserved amino acids, : denotes interchangeable amino acids properties, and · denotes amino acids with similar properties.

Figure S3 ChIP was carried out on formaldehyde fixed chromatin complexes from passage 1 day 34 primary chorionic girdle trophoblasts, using an antihuman glial cells missing 1 (GCM1) antibody for immunoprecipitation. Binding of GCM1 to predicted GCM1-binding sites was determined using PCR and primers designed to detect sites, 1–4 and two control sites that were not predicted GCM1 binding sites within 500 bp (*n* = 2 conceptuses). Only sites 1–3 and one control site were taken forward to quantitative RT-PCR shown in Figure 3A.
