# Supplementary figures and images for: Glial Cells Missing 1 Regulates Equine Chorionic Gonadotrophin Beta Subunit via Binding to the Proximal Promoter
Source: Front Endocrinol (Lausanne). 2018 Apr 26;9:195. doi: 10.3389/fendo.2018.00195 (PMC5932191; doi:10.3389/fendo.2018.00195)

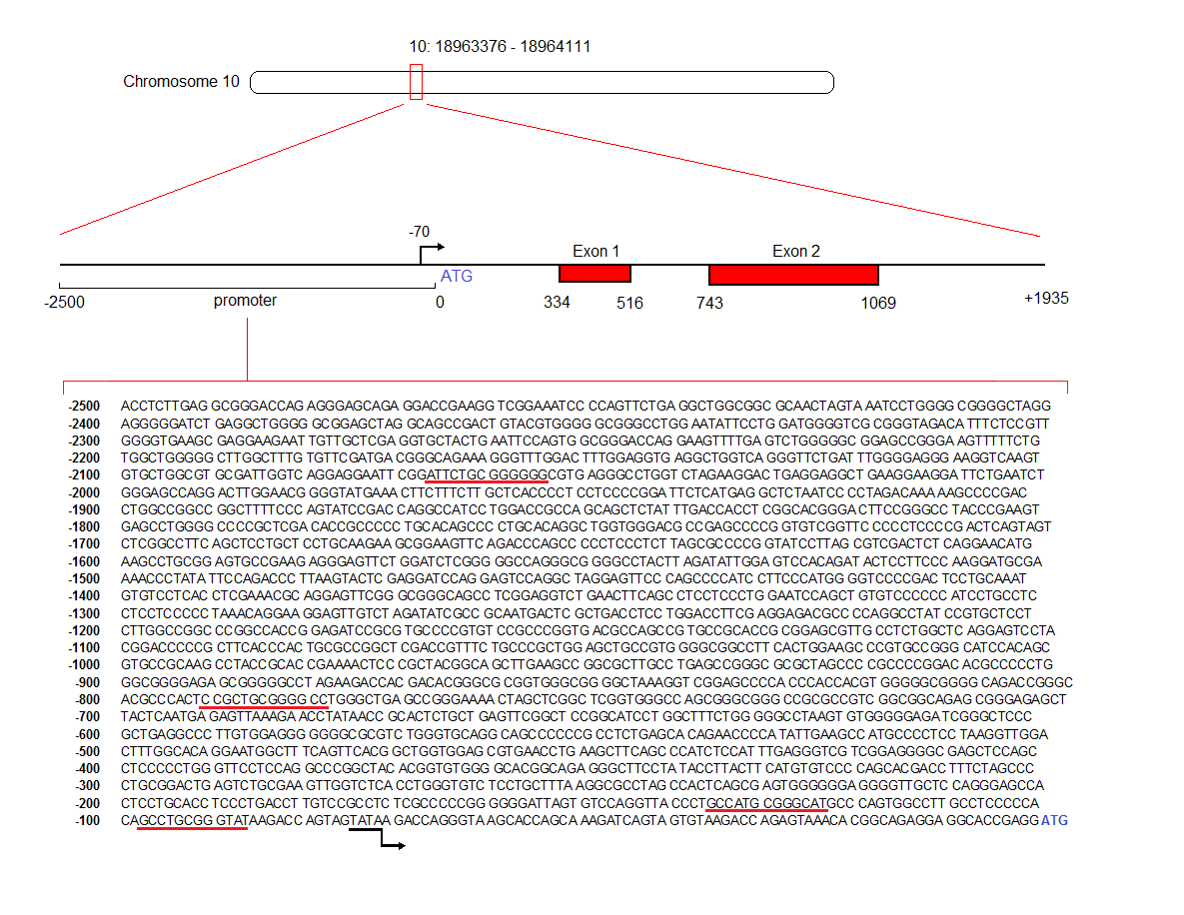

Supplement: Figure S1 — The CGB promoter contains four possible glial cells missing 1 (GCM1)-binding sites. The 2,500-bp LHB promoter sequence was obtained from ENSEMBL genome browser and Match software was used to identify GCM1 consensus binding sites with a core sequence match of 1 and matrix similarity >0.8. The four consensus-binding sites (underlined in red) for GCM1 are located at positions (1) −87 to −98 (2) −124 to −136 (3) −779 to −791 and (4) −2055 to −2067, relative to the translational start site. Arrow depicts transcriptional start site at −70 bp, and ATG depicts translational start site. Numbers on diagram are relative to translational start site. [file image_1.TIFF]

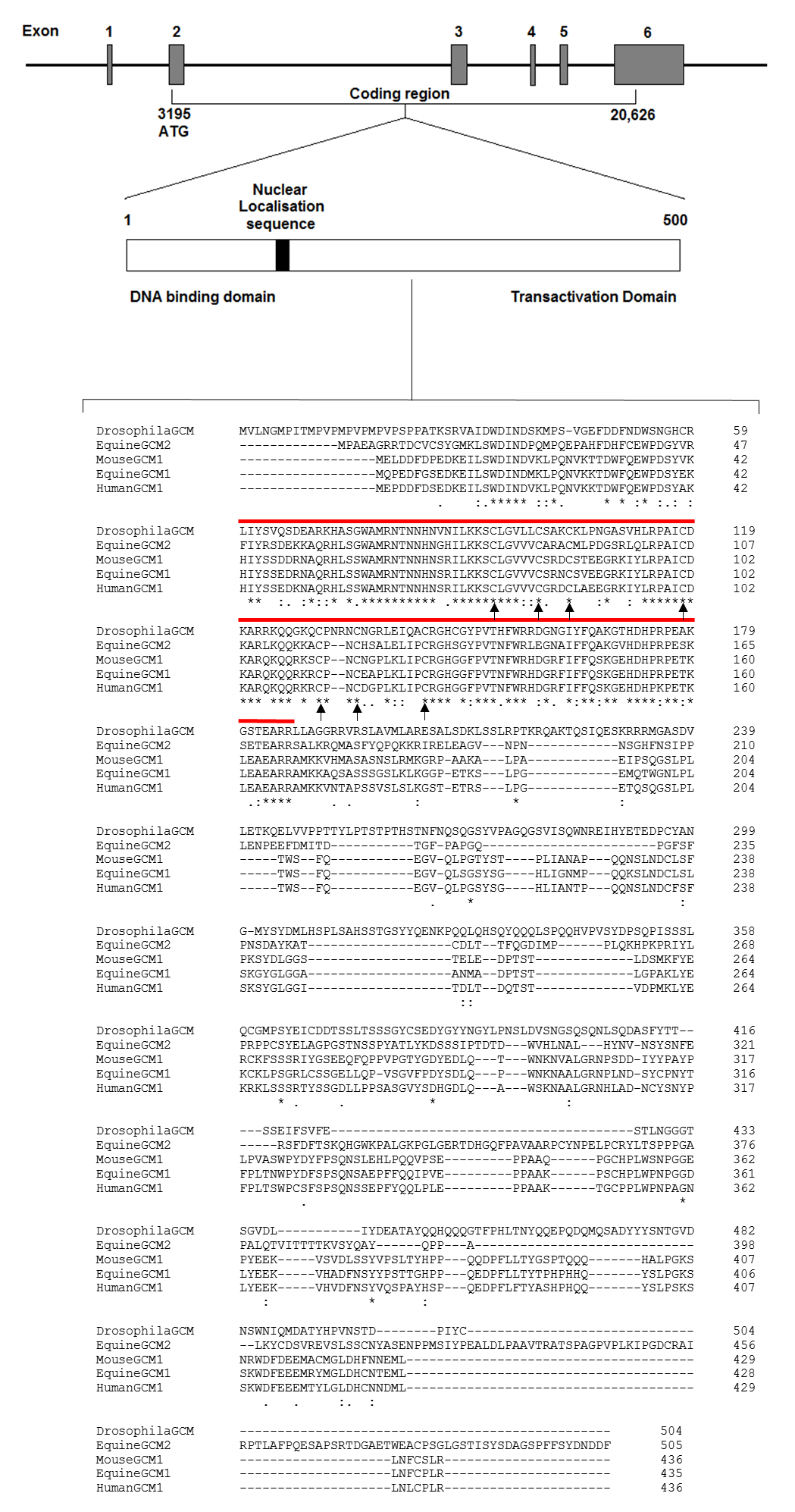

Supplement: Figure S2 — Structure of the glial cells missing 1 (GCM1) family. Shown is human GCM1, located on chromosome 6. The coding region of glial cells missing spans exons 2 to 6 in the genomic sequence. Nucleotide position numbers show start and finish of coding region. The protein consists of a conserved N-terminal DNA binding domain, underlined in red on the cross-species alignments, a nuclear localization sequence and two C-terminal transactivation domains. The C-terminal region shows poor species conservation. Seven conserved cystein residues are shown with black arrows. * denotes conserved amino acids, : denotes interchangeable amino acids properties, and · denotes amino acids with similar properties. [file image_2.JPEG]

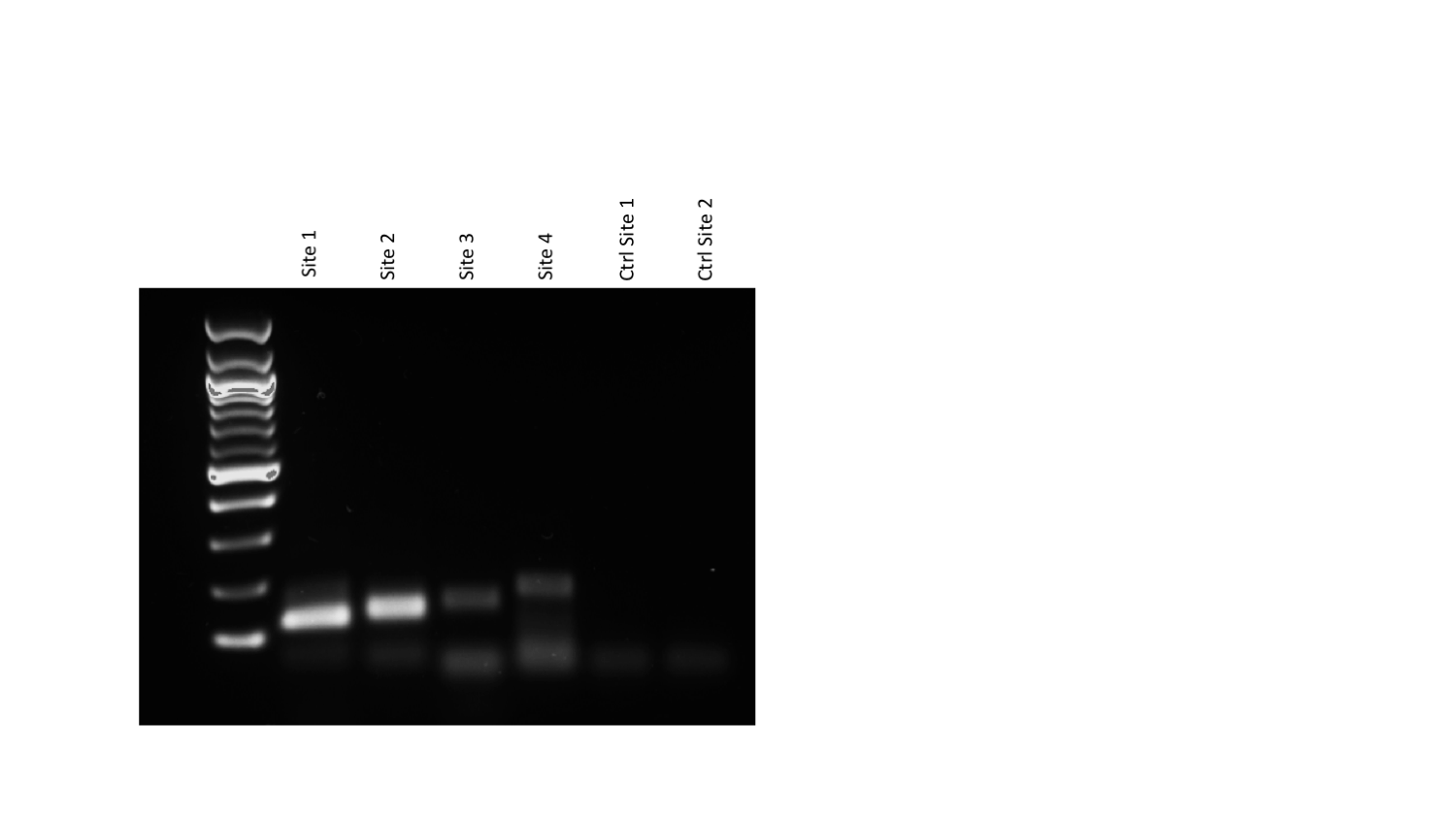

Supplement: Figure S3 — ChIP was carried out on formaldehyde fixed chromatin complexes from passage 1 day 34 primary chorionic girdle trophoblasts, using an antihuman glial cells missing 1 (GCM1) antibody for immunoprecipitation. Binding of GCM1 to predicted GCM1-binding sites was determined using PCR and primers designed to detect sites, 1–4 and two control sites that were not predicted GCM1 binding sites within 500 bp (n = 2 conceptuses). Only sites 1–3 and one control site were taken forward to quantitative RT-PCR shown in Figure 3A. [file image_3.TIFF]
