# Supplementary material for: Glial Cells Missing 1 Regulates Equine Chorionic Gonadotrophin Beta Subunit via Binding to the Proximal Promoter
Source: Front Endocrinol (Lausanne). 2018 Apr 26;9:195. doi: 10.3389/fendo.2018.00195 (PMC5932191; doi:10.3389/fendo.2018.00195)
Supplement: Supplementary file 4 [file data_sheet_1.DOCX]

**Supplementary Data**

**Supplementary Table 1 Cloning primers for amplification of *eCGβ* promoter inserts.** Shown are promoter inserts to be amplified, primers used, primer sequences (tagged with 5’ MluI and 3’ BglII), annealing temperature (Tm) for primer pairs, expected product size and primer supplier.

**Supplementary Table 2 Primers used for qRT-PCR.** Shown are genes amplified, primer names, primer sequences, annealing temperature of primer pairs (Tm), expected product size and primer supplier.

**Supplementary Table 3: Primers used for ChIP qRT-PCR.**

| **GCM1 site** | **Primer** | **Primer Sequence** | **Tm** | **Product Size** | **Manufacturer** |
| --- | --- | --- | --- | --- | --- |
| 1 | chp GCM1 1 Fw | AGTGGCCTTGCCTCCCCCA | 65°C | 119 bp | Eurofins Genomics |
|  | chp GCM1 1 Rev | CCTCGGTGCCTCCTCTGC |  |  | Eurofins Genomics |
| 2 | chp GCM1 2 Fw | GGGGTTGCTCCAGGGAGCC | 67°C | 119 bp | Eurofins Genomics |
|  | chp GCM1 2 Rev | TGGGGGAGGCAAGGCCACT |  |  | Eurofins Genomics |
| 3 | chp GCM1 3 Fw | GAGCCCCACCCACCACGTGG | 65°C | 138 bp | Eurofins Genomics |
|  | chp GCM1 3 Rev2 | AGCTCTCCCGCTCTGCC |  |  | Eurofins Genomics |
| Ctrl | chp C5 Fw | CTGCTCTACCATTATGTTG | 56°C | 131 bp | Eurofins Genomics |
|  | chp C5 Rev | TCTCAAAGTCCAGGATCTC |  |  | Eurofins Genomics |
